# Analysis on associated factors of uncontrolled hypertension among elderly hypertensive patients in Southern China: a community-based, cross-sectional survey

**DOI:** 10.1186/1471-2458-14-903

**Published:** 2014-09-02

**Authors:** Li Yang, Xiaoling Xu, Jing Yan, Wei Yu, Xinhua Tang, Haibin Wu, Christy L Parkin

**Affiliations:** Zhejiang provincial center for cardio-cerebrovascular diseases control and prevention, Zhejiang hospital, 12 Lingyin Road, Hangzhou, Zhejiang 310013 China; Zhejiang provincial center for disease control and prevention, Hangzhou, 310051 China; Sino-US Diabetes Center, Zhejiang Hospital, 12 Lingyin Road, Hangzhou, Zhejiang 310013 China

**Keywords:** Control, Elderly hypertensives, Cross-sectional, Associated factors

## Abstract

**Background:**

The prevalence of hypertension in China has risen dramatically in recent decades, but it is not well understood if hypertension is adequately controlled in the elderly population in Southern China. A provincial survey was performed in order to estimate the prevalence of hypertension control and the associated factors in the elderly population.

**Methods:**

A cross-sectional survey was conducted in 6 community health service centers across 12 counties in Southern China from October 2010 to April 2011. Recruitment included a total of 10644 hypertensive subjects aged 60 or older. Basic lab tests and physical examinations were administrated on each subject. In addition, each subject completed a standardized questionnaire.

**Results:**

The 10644 participants (mean age of 70.3 years) included 5527 women (51.9%), 5117 men (48.1%), 3148 overweight subjects (29.57%), 846 (7.95%) obese subjects, 1654 smokers (15.54%) and 1750 consumers of alcohol (16.44%). The control, treatment and awareness of hypertension were 44.6%, 50.3% and 46.3%, respectively. Most treated hypertensives (68.57%) used combination therapy of antihypertensive medications, and those using long-acting antihypertensive medications had a higher rate of adequately controlled hypertension. Results showed that elderly, living in rural and suburban areas, low education level, family history of hypertension, smoking, excessive salt consumption, lack of physical activity, overweight, obese and diabetes were associated with uncontrolled hypertension.

**Conclusions:**

Lack of adequate hypertension control is relatively high among the elderly in Southern China. Hypertension awareness and early treatment are needed in this population, especially among suburban population, as well as adopting appropriate antihypertensive medication therapy and healthy lifestyles.

**Electronic supplementary material:**

The online version of this article (doi:10.1186/1471-2458-14-903) contains supplementary material, which is available to authorized users.

## Background

Cardiovascular disease (CVD) is one of the leading causes of death worldwide, and it is enormous burden in developed and developing countries [1]. As one of the most important risk factors of CVD, hypertension accounts for approximately 50% of coronary heart disease and 67% for the cerebrovascular disease burden worldwide [2]. Recently, a survey demonstrated a decreasing trend of hypertension in western countries, but an increasing trend among Southeast Asia and Oceania [3]. In China, hypertension has ranked first as a risk factor of CVD, and many associated complications, such as stroke, heart and renal diseases, which are major causes of mortality [4]. According to the 2002 National Nutrition and Health Survey (NNHS), the prevalence of hypertension in adult Chinese population of 60 years or older was 49.1%, and the rates of awareness, treatment and control were 37.6%, 32.2%, and 7.6%, respectively [5].

Zhejiang province is located in Southern China, it has an aging population (60 years or older) of approximately 7.5 million in 2011 which accounts for 13.39% of the total population. As one of the most rapidly developing provinces, dramatic social and economic changes, including rapid urbanization has occurred in Zhejiang over the past 2 decades. These factors, in part, contributed to the inadequate control of hypertension [6]. However, little is known about whether hypertension is adequately controlled among the elderly in Southern China. A survey was conducted with 10644 elderly hypertensive patients living in Zhejiang province to study awareness, treatment and control of hypertension, and investigate the associated factors of uncontrolled hypertension.

## Methods

### Sampling

This study conducted in Zhejiang province was a cross-sectional survey administrated in 6 community health centers across 12 counties. A multistage stratified random cluster sampling was used to include the subjects. In the first stage, 12 administrative districts were divided into Type 1 urban districts, Type 2 suburban districts and Type 3 rural districts based on economic levels. From each of these three groups, a district was systematically selected. The first stage included 3 districts. Two communities were randomly chosen from each district for the second stage. Subjects aged 60 years or more with hypertension living in the selected communities were invited to participate for the third stage. The cluster intra-group correlation intraclass correlation coefficient (ICC) is 0.02. Ethics approval was obtained from the Ethics Committee of Zhejiang Hospital, and all participants signed an informed consent.

In the period from October 2010 to April 2011, 11000 subjects with hypertension older than 60 were recruited. In the final analysis, 356 subjects were excluded because of lack of information or low compliance. A total of 10644 subjects were included in the analysis (response rate was 96.7%); there were no heterogeneities between the excluded 356 patients and the included 10644 patients. The response rates of the 3 different clusters were 96.36%, 94.37% and 98.78% respectively, there were no significant differences (*P* = 0.34).

### Field survey and quality control

Data collection of this field survey included a questionnaire interview, physical examinations and biochemical examinations. A standardized questionnaire was administered by trained general practitioners in community public health service centers during a face-to-face individual interview. The questionnaire included demographic information such as age, areas of residence, education and health behaviors such as history of smoking, alcohol consumption, diet and physical activity. Physical examinations performed by trained nurses included height, weight, waist circumference, blood pressure (BP) and biochemical examinations including fasting plasma glucose (FPG) and lipid profile.

Weight and height were measured with subjects standing without shoes and wearing light clothing. Participants stood upright with the head in Frankfort plane for height measurement. Height was recorded to the nearest 0.5 cm, and weight was recorded to the nearest 100 g. Body-mass-index (BMI) was calculated as weight in kilograms over height in meters squared [weight(kg)/(height(m))^2^] [7]. Waist circumference was measured at the level of the iliac crest using a non-elastic tape measure.

Blood specimens were collected in vacuum tubes after at least 10 hours of overnight fasting, for the measurement of plasma glucose concentration and serum concentrations of total cholesterol and triglycerides. Plasma glucose was measured with the glucose oxidase method. Lipids were measured using Beckman auto analyzer at the biochemistry laboratory of Zhejiang Hospital [7].

BP measurements were performed using standardized mercury sphygmomanometers in this study. Subjects were advised to avoid consuming alcohol or tobacco, ingesting tea or coffee, or engaging in exercise for at least 30 minutes before BP measurement. In addition, subjects were asked to rest for at least 15 minutes before BP testing in a quiet room. The average of two measurements taken 5 minutes apart will be used, but if systolic BP (SBP) measurements differed by 10 mmHg or greater, or diastolic BP (DBP) differed by 5 mmHg or greater, a third measurement will be taken. In this instance the average of the two closest SBP and two closest DBP measurements will be used to define the BP [8].

Knowledge about hypertension was tested by questionnaire including the normal blood pressure values, and some healthy lifestyles related to hypertension. Awareness of hypertension was defined as subjects knowing their own blood pressure values. Treatment of hypertension was defined as taking a prescribed medicine for management of hypertension.

### Definition

According to the Chinese Guidelines on Prevention and Control of Hypertension [9], hypertension is defined as SBP ≥ 140 mmHg and/or DBP ≥ 90 mmHg, or self-reported treatment of hypertension with antihypertensive medication. Pre-hypertension is defined as 120 mmHg ≤ SBP ≤ 139 mmHg and/or 80 mmHg ≤ DBP ≤ 89 mmHg, Stage II hypertension is defined as 160 mmHg ≤ SBP ≤ 179 and/or 100 mmHg ≤ DBP ≤ 109 mmHg, and stage III hypertension is defined as SBP ≥ 180 mmHg and/or DBP ≥ 110 mmHg. According to the recent JNC 8 Hypertension Guidelines, a BP goal of less than 150/90 mmHg for hypertensive persons aged 60 years or older is defined as control of hypertension [10].

According to the Chinese criteria, overweight is defined as 24 kg/m^2^ ≤ body mass index (BMI) < 28 kg/m^2^; obesity was defined as BMI ≥ 28 kg/m^2^[11]. Fasting plasma glucose (FPG) is classified as impaired fasting glucose (IFG) with 6.1 mmol/L ≤ FBG ≤ 6.99 mmol/L, and diabetes mellitus (DM) as FBG ≥ 7.0 mmol/L [11]. Serum triglycerides (TG) are classified as moderately high TG (1.7 mmol/L ≤ TG ≤ 2.25 mmol/L); high TG (TG ≥ 2.26 mmol/L). Serum total cholesterol (TC) is categorized as moderately high TC (5.18 mmol/L ≤ TC ≤ 6.19 mmol/L); high TC (TC ≥ 6.22 mmol/L). Low serum high-density lipoprotein cholesterol (HDL-C) are defined as HDL-C < 1.04 mmol/L [11].

Subjects who smoked one cigarette or more per day for over 6 months were defined as smokers, and alcohol drinkers were assessed by asking subjects whether they had consumed more than once every week in the last 12 months. Subjects who consumed more than 10 grams salt per day for over 6 months were defined as excessive salt users.

### Statistical analysis

The sampling design including stratification, clustering and sampling weights (accounting for differential probabilities of selection and also a post-stratification on gender, age) was taken into account in all estimates and analyses using the specific SAS commands.

Epidata 3.0 was used for data entry and validation and SAS 9.2 for data management and analysis. Socio-demographic characteristics, physical measurements and hypertension status of participants were summarized using frequencies (percentages) or means and standard deviations. Mean values and proportions were compared by the Student’s t-test and chi-square test, respectively. The trends in prevalence of hypertension associated factors across categories were analyzed using chi-square test. And the strength of associations of socio-demographic associated factors of hypertension was assessed for binary response variable (uncontrolled hypertension yes/no) by Odds-Ratios (OR) estimated in logistic regression models. Crude associations were first assessed using univariate models, then associations were assessed using multivariate models, and 95% confidence intervals were calculated. The final model was evaluated by goodness of fit. Significance level was set at p < 0.05 for all hypothesis tests.

## Results

### Characteristics of the studied population

The 10644 participants (mean age of 70.33 years) included 5527 women (51.93%), 5117 men (48.1%), 3148 overweight subjects (29.57%), 846 (7.95%) obese subjects, 1654 smokers (15.54%) and 1750 drinkers (16.44%). Men, compared with women, had a lower BMI, FBG, TG, TC and higher level of education; reported significantly higher proportions of current smoking, alcohol use, and had a lower prevalence of overweight and obesity (Table 1). The demographic characteristics of participants in different clusters such as gender, age and education level were showed in the Additional file 1.

**Table 1:** **Distribution of socio demographic factors among participants, by gender (n = 10644)**

Variables	Men (n = 5117)	Women (n = 5527)	P-value
**Age (years)**	70.4 ± 7.3	70.3 ± 7.6	0.008
**BMI (kg/m2)**	23.6 ± 2.9	24.9 ± 3.4	0.003
**Blood pressure (mm Hg)**			
Systolic	142.7 ± 15.7	142.6 ± 15.6	0.77
Diastolic	84.38 ± 8.96	83.6 ± 8.8	0.19
**Area of residence, n (%)**			
Urban	1468 (28.7)	1542 (27.9)	0.37
Suburban	2144 (41.9)	2337 (42.3)	
Rural	1505 (29.4)	1648 (29.8)	
**Education, n (%)**			
College or higher	271 (5.3)	116 (2.1)	<0.001
Middle	1039 (20.3)	668 (12.1)	
Primary	2446 (47.8)	1766 (31.9)	
Illiterate	1361 (26.6)	2977 (53.9)	
**Family history of hypertension, n (%)**	951 (18.6)	995 (18.0)	0.08
**Smoker, n (%)**	1229 (24.0)	425 (7.7)	<0.001
**Alcohol intake, n (%)**	1298 (25.4)	452 (8.2)	<0.001
**Overweight, n (%)**	1470 (28.7)	1678 (30.4)	0.01
**Obesity, n (%)**	315 (6.2)	531 (9.6)	0.003
**Biochemical measurements**			
FBG (mmol/L)	5.32 ± 1.14	5.51 ± 1.33	0.002
TG (mmol/L)	1.51 ± 0.93	1.75 ± 1.08	0.001
TC (mmol/L)	4.48 ± 1.00	4.60 ± 1.06	0.03
HDL-C (mmol/L)	1.45 ± 0.75	1.40 ± 0.58	0.06

### Control of hypertension

Control of hypertension for participants of this survey is presented in Table 2. Of the 10644 hypertensive patients aged 60 years or older, 4747 achieved a BP under 150/90 mmHg. The control rate of hypertension was 44.6% according to the JNC 8 Guidelines, the control rate was 34.5% if the BP goal of less than 140/90 mmHg was defined as control of hypertension. And 27.3% participants had BP of more than 160/100 mmHg (stage II and stage III hypertension).

**Table 2:** **Control of hypertension among elderly hypertensive patients in Southern China in 2011**

Variables	Normal	Pre-hypertension	Hypertension		Control rate (%)	P-value ^1^
			Stage II	Stage III		
**All subjects, n (%)**	138 (1.3)	3532 (33.2)	2885 (27.1)	25 (0.2)	44.6	
**Sex**						
Women	66 (1.2)	1895 (34.3)	1470 (26.6)	7 (0.1)	46.8	0.003
Men	72 (1.4)	1637 (32.0)	1415 (27.6)	18 (0.3)	42.6	
**Age, n (%)**						
60-69 years	77 (1.4)	1817 (34.3)	1309 (24.7)	11 (0.2)	50.8	<0.001
70-79 years	45 (1.2)	1235 (31.5)	1158 (29.5)	9 (0.2)	40.7	
≥80 years	16 (1.1)	430 (30.2)	478 (33.5)	5 (0.4)	37.6	
**Area of residence, n (%)**						
Urban	105 (3.3)	1119 (35.5)	663 (21.0)	6 (0.2)	51.1	<0.001
Suburban	55 (1.2)	979 (21.8)	1511 (33.6)	13 (0.3)	31.4	
Rural	23 (0.8)	708 (23.5)	898 (29.8)	6 (0.2)	34.2	
**Education, n (%)**						
College or higher	5 (1.3)	131 (35.6)	98 (26.7)	1 (0.2)	49.2	<0.001
Middle	23 (1.4)	557 (33.7)	440 (26.6)	2 (0.1)	45.2	
Primary	33 (0.8)	1191 (29.2)	1113 (27.3)	9 (0.2)	40.2	
Illiterate	34 (0.8)	1038 (24.7)	1297 (30.8)	13 (0.3)	33.7	
**Family history of hypertension, n (%)**	62 (1.2)	2010 (39.3)	1006 (19.7)	5 (0.1)	42.8	
**Smoker, n (%)**	34 (1.0)	1016 (30.4)	968 (28.9)	5 (0.1)	38.2	
**Drinker, n (%)**	37 (1.1)	1021 (30.4)	961 (28.6)	5 (0.1)	39.4	
**Overweight, n (%)**	28 (0.8)	998 (30.2)	984 (29.8)	4 (0.1)	37.4	
**Obesity, n (%)**	31 (1.1)	751 (26.0)	898 (31.1)	7 (0.2)	33.3	
**FBG (mmol/L)**						
Normal	67 (1.0)	2527 (36.4)	1856 (26.8)	6 (0.1)	50.6	<0.001
IFG	7 (1.1)	178 (28.2)	194 (30.7)	9 (1.4)	40.4	
DM	2 (0.4)	135 (25.9)	161 (30.9)	10 (1.9)	36.5	
**TG (mmol/L)**						
Normal	50 (1.1)	1516 (33.3)	1159 (25.4)	6 (0.1)	48.7	<0.001
Slightly high	14 (1.1)	324 (25.0)	452 (34.9)	9 (0.5)	36.4	
High	6 (0.6)	256 (23.7)	360 (33.4)	10 (0.9)	31.4	
**TC (mmol/L)**						
Normal	58 (1.1)	1542 (27.9)	1639 (29.6)	9 (0.2)	45.9	0.003
Slightly high	12 (0.9)	371 (27.1)	422 (30.8)	6 (0.4)	42.2	
High	1 (0.3)	114 (31.7)	91 (25.3)	10 (2.8)	37.4	
**HDL-C (mmol/L)**						
Normal	86 (1.2)	1981 (26.9)	2174 (29.5)	35 (0.5)	43.3	0.004
Low	93 (1.3)	1782 (24.2)	2144 (29.1)	14 (0.2)	40.8	

^1^P-value: Comparisons among control rates by chi-square test.

Compared with men, women had slightly higher control rate of hypertension (46.8% *vs.* 42.6%, *P* = 0.003), and the control rate decreased with older age (*P* < 0.001). Participants living in suburban areas had lower control rates of hypertension compared with living in rural and urban areas (31.4% *vs.* 34.2% *vs.* 51.1%, P < 0.0001). The control rate was higher among those with lower education levels and those with higher BMI’s (all P < 0.001). The rate of uncontrolled hypertension was increased with age and BMI, and decreased with the level of education. Smokers, drinkers, and the groups with family history of hypertension, lower HDL-C, high FBG or TC had lower control rate of hypertension (Table 2).

Prevalence of stage II and stage III hypertension differed by gender. Overall they were higher for men (all P < 0.001), and the prevalence of stage II and stage III hypertension increased with age; there were statistically significant (X2 = 47.92, *P* = 0.02).

### Awareness and treatment of hypertension

**Figure 1:**
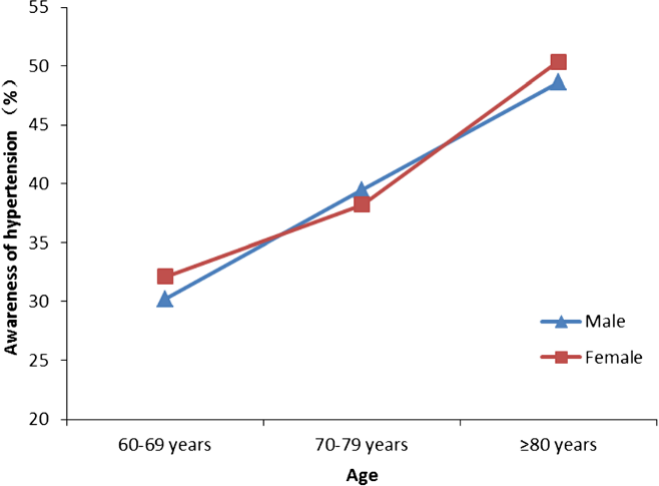
**The awareness of hypertension among elderly hypertensive patients in Southern China in 2011.**

**Figure 2:**
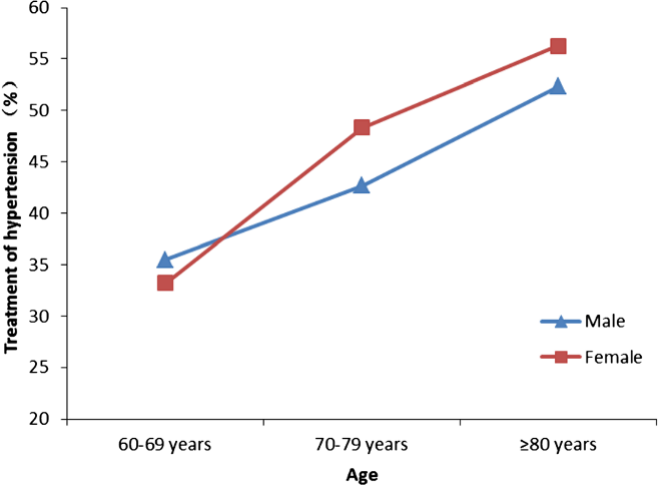
**The treatment of hypertension among elderly hypertensive patients in Southern China in 2011.**

Awareness of hypertension was defined as subjects knowing their own blood pressure values. In the 10644 hypertensive subjects, 4924 (46.27%) were aware of their normal blood pressure values, 5357 (50.33%) were taking antihypertensive drugs. Men, compared with women, had slightly lower rates of awareness (42.05% *vs.* 48.69%, *P* = 0.003) and treatment (47.51% *vs.* 52.06%, *P* < 0.001). In men as well as women, the awareness and treatment rates of hypertension were higher with older age (all *P* < 0.001, Figures 1 and 2).

### Antihypertensive medications among treated patients

Table 3 showed the use of antihypertensive medications according to areas among the 5357 treated hypertensive patients. The results showed that 68.57% of treated hypertensives used combination therapy, and 32.36% chose monotherapy. Urban participants, compared with suburban and rural participants, more frequently used a combination of antihypertensive drugs including single pill combination treatment (*P* < 0.001, Table 3). The control rate was higher among subjects using combination of antihypertensive medications compared with that of using monotherapy (62.2% *vs.* 52.4%, *P* < 0.001).

**Table 3:** **Prevalence of use of commonly prescribed classes of anti-hypertension medications among hypertensive participants (n,%)**

Medication	Urban (n = 3010)	Suburban (n = 4481)	Rural (n = 3153)	All (n = 10644)	P-value ^1^
**Monotherapy**	764 (25.4)	1675 (37.4)	1006 (31.9)	3445 (32.4)	<0.001
DIU	65 (2.2)	443 (9.9)	139 (4.4)	647 (6.1)	0.001
ACEI/ARB	157 (5.2)	313 (7.0)	174 (5.5)	644 (6.1)	0.003
BB	22 (0.7)	57 (1.3)	49 (1.5)	128 (1.2)	0.04
CCB	520 (17.3)	862 (19.2)	644 (20.4)	2026 (19.0)	0.04
**Combination**	2246 (74.6)	2906 (62.6)	2147 (68.1)	7299 (68.6)	<0.001
Fixed-dose combination	1426 (47.4)	1748 (39.0)	1578 (50.1)	4752 (44.6)	<0.001
Single pill combination	863 (28.7)	946 (21.1)	712 (20.6)	2521 (23.7)	0.002
Free combination	43 (1.4)	212 (4.7)	143 (4.5)	398 (3.7)	0.012

^1^P-value: Comparisons among urban, suburban and rural subjects by chi-square test.

### Factors associated with uncontrolled hypertension

Logistic regression models including crude model and adjusted model were used to assess factors associated with uncontrolled hypertension, respectively. The model adjusted for demographic factors such as gender, age, areas, showed that elderly, living in suburban and rural areas compared with living in urban areas, low level of education, family history of hypertension, smoking, alcohol use, excessive salt consumption, lack of physical activity, overweight and obesity and high FPG were associated with uncontrolled hypertension (Table 4).

**Table 4:** **Factors associated with uncontrolled hypertension among elderly hypertensive patients in Southern China in 2011 analyzed by crude and adjusted regression model**

Variables	Total (n = 10644)		Men (n = 5117)		Women (n = 5527)	
	COR ^1^ (95%CI) ^3^	AOR ^2^ (95%CI) ^3^	COR ^1^ (95%CI) ^3^	AOR ^2^ (95%CI) ^3^	COR ^1^ (95%CI) ^3^	AOR ^2^ (95%CI) ^3^
**Age (ref: 60–69 years)**						
70-79 years	1.5 (1.3-1.9)	1.6 (1.4-2.2)	1.7 (1.3-1.9)	1.9 (1.6-2.7)	1.5 (1.0-1.8)	1.7 (1.2-2.4)
≥ 80 years	1.8 (1.5-2.1)	1.9 (1.6-2.6)	1.8 (1.5-2.1)	2.2 (1.5-3.3)	1.4 (1.0-1.6)	1.6 (1.2-1.9)
**Area of residence (ref: Urban)**						
Suburban	1.5 (1.2-1.6)	1.6 (1.1-1.9)	1.4 (1.0-1.9)	1.7 (1.2-2.5)	1.1 (1.1-1.6)	1.4 (1.0-2.0)
Rural	1.3 (1.1-1.6)	1.4 (1.1-2.1)	1.3 (1.0-2.0)	1.5 (1.1-2.2)	1.0 (1.0-1.8)	1.3 (1.0-1.8)
**Education (ref: College or higher)**						
Middle	1.7 (1.4-2.3)	1.3 (0.8-2.0)	1.6 (1.3-2.3)	1.2 (0.7-1.8)	1.7 (1.3-2.5)	1.3 (0.9-1.9)
Primary	2.0 (1.5-2.7)	1.5 (1.2-1.9)	2.0 (1.7-2.6)	1.9 (1.5-2.6)	1.9 (1.5-2.9)	1.4 (0.9-2.1)
Illiterate	2.3 (1.7-2.8)	2.1 (1.6-2.7)	2.4 (1.8-3.0)	2.9 (2.1-4.1)	2.2 (1.6-2.8)	1.7 (1.3-2.3)
**Family history of hypertension (ref: No)**	1.8 (1.5-2.6)	1.2 (1.0-1.3)	1.5 (1.0-2.0)	1.6 (1.1-2.4)	1.9 (1.1-2.2)	2.0 (1.2-2.6)
**Smoker (ref: Nonsmoker)**	2.7 (2.1-4.1)	2.8 (1.6-3.9)	3.4 (1.2-2.2)	3.8 (2.1-4.5)	2.3 (1.2-3.8)	2.5 (1.4-3.8)
**Drinker (ref: Nondrinker)**	2.5 (1.5-3.9)	2.0 (0.9-3.1)	2.7 (1.6-4.1)	2.1 (1.2-3.3)	2.2 (1.2-3.3)	1.9 (0.8-2.6)
**Excessive salt (ref: No)**	1.3 (1.0-1.6)	1.5 (1.1-2.4)	1.0 (0.9-1.2)	1.2 (0.9-2.1)	0.9 (0.5-1.1)	0.6 (0.2-1.1)
**Physical activity < 2 hours/week (ref: No)**	0.6 (0.1-0.9)	0.8 (0.0-0.9)	0.7 (0.0-0.9)	0.8 (0.5-0.9)	0.6 (0.1-1.1)	0.7 (0.2-1.2)
**BMI (ref: Normal)**						
Overweight	2.5 (2.0-3.6)	2.3 (1.5-4.0)	2.1 (1.8-2.5)	2.4 (1.2-3.2)	2.4 (2.1-2.8)	2.5 (1.5-3.5)
Obesity	2.9 (1.9-3.8)	3.3 (2.9-4.3)	2.4 (1.9-3.0)	3.0 (1.3-3.9)	2.5 (2.1-3.1)	2.6 (1.4-3.8)
**FPG (ref: Normal)**						
IFG	2.4 (1.5-3.2)	2.5 (1.2-3.2)	2.5 (1.5-3.2)	2.6 (1.9-3.6)	2.4 (1.6-3.3)	3.2 (0.8-4.0)
DM	2.7 (1.4-4.2)	1.2 (1.0-2.4)	3.1 (2.3-4.0)	3.3 (2.3-4.2)	2.8 (1.9-4.3)	2.5 (1.2-3.2)
**TG (ref: Normal)**						
Slightly high	2.6 (1.3-2.9)	2.1 (0.8-3.2)	2.2 (1.3-3.1)	2.3 (0.9-3.1)	2.5 (1.5-2.9)	2.6 (1.9-3.5)
High	2.8 (1.4-3.2)	2.5 (0.9-2.9)	2.5 (1.2-3.3)	2.6 (1.6-3.5)	2.7 (1.5-3.2)	2.8 (2.0-3.6)
**TC (ref: Normal)**						
Slightly high	0.6 (0.3-1.1)	0.5 (0.0-1.3)	0.5 (0.1-0.8)	0.6 (0.2-0.9)	0.6 (0.0-1.6)	1.1 (1.0-1.3)
High	0.7 (0.4-0.9)	0.2 (0.0-1.6)	0.9 (0.6-1.2)	1.0 (0.6-1.5)	0.8 (0.2-0.9)	0.7 (0.0-0.9)
**Low HDL-C (ref: Normal)**	0.6 (0.4-0.9)	0.7 (0.1-1.1)	1.3 (1.0-1.8)	1.1 (0.8-2.1)	1.2 (1.0-1.9)	1.2 (1.0-1.8)

^1^COR: Crude odds ratio in the model with unadjusted association.

^2^AOR: Adjusted odds ratios in the model with multivariate models adjusted by age group and area of residence.

^3^95% CI, 95% confidence interval.

In the adjusted model, the associations between uncontrolled hypertension and smoking, alcohol use, lack of physical activity and higher FPG were more pronounced in men than women. The associations between uncontrolled hypertension and TG and TC were less pronounced for men (Table 4).

## Discussion

Hypertension is a major independent risk factor for cardiovascular disease and stroke. It has been estimated that more than one quarter of the world adult population had hypertension in the year 2000, and that this would increase to 29% by the year 2025 [7]. Zhejiang province has a rapidly aging population with the percentage of older people increasing each year, which is an important factor associated with hypertension, special attention should be given to the elderly patients.

Our study indicates that the rate of controlled hypertension among patients older than 60 in Southern China (34.5%) is higher than that in Northern parts of China (27.5%) and lower than developed countries such as the United States (54.9%) [12, 13], probably due to the different living styles and dietary habits [14].

Although hypertension awareness, treatment and control among elderly patients have markedly improved compared with the elderly subgroup (≥60 years of age) of the 2002 NNHS [15], they were higher than the study surveyed in Beijing [16] and some developed countries [17–19], the control of hypertension was still unsatisfactory among hypertensive patients.

Most patients in our study used combination therapy of antihypertensive medications, those using long-acting antihypertensive medications had more higher control rate; and those using a monotherapy of short-acting antihypertensive medications had lower control rate. There were more patients using inappropriate antihypertensive medications in suburban and rural areas, possibly due to the lack of qualified physicians in these areas.

Old age, high FBG, overweight, obesity and family history of hypertension are traditional associated factors of hypertension [20–22]. Some reports have shown that excessive alcohol use and tobacco consumption are also important causes of hypertension [23–25]; results in this study provide additional support to these hypotheses. Adjusted odds ratios (AOR) were greater in the groups with higher age and lower education, which is similar with some other studies [26–28]. These results also showed that excessive use of salt and lack of physical activity were associated with higher rates of uncontrolled hypertension. These further confirmed some of the previous reports [29–32], but were in contrast with the Awoke study [33].

Compared with living in urban areas, living in suburban and rural areas are associated factors of uncontrolled hypertension. China is in a rapid urbanization period and in the past few decades, a large number of rural people, such as our study subjects, immigrated to cities or were locally urbanized. They often travel to cities for work and come back home during weekends, and typically have more stress related issues and unhealthy lifestyles that result in uncontrolled hypertension. More effective primary prevention measures should be made in these areas to address the rise in hypertension.

Some limitations needed to be mentioned in this study. Firstly, our study was a cross-sectional survey, which made it difficult to establish a cause-and-effect relationship between associated factors and uncontrolled hypertension. Second, we did not include the socioeconomic status, such as personal or family income, which may be associated with the prevalence of hypertension and lack of adequate control rate. Despite these limitations, the strengths of this study included providing regional representative data and evidence-based references for the control and management of hypertension at a population level.

## Conclusions

Uncontrolled hypertension is highly prevalent in elderly hypertensive patients in Southern China. Although the rates of control, treatment and awareness have increased significantly since 2002, many of the hypertensive patients are unaware of their condition, and the rate of control and treatment remain relatively low, most likely due to the inappropriate use of antihypertensive medications and unhealthy lifestyles. Innovative strategies and efforts to improve the management of hypertension are needed, including the appropriate use of antihypertensive drugs and the intervention of factors associated with hypertension, such as smoking, drinking alcohol, excessive salt consumption, obesity, etc. More population-based strategies to control and prevent hypertension, including conducting community-based intervention programs, carrying out primary prevention policies and public health services are needed to address this serious problem.

## Electronic supplementary material

Additional file 1: **Distribution of socio demographic factors among participants, by clusters (n = 10644).** (DOC 42 KB)

## Abbreviations

CVD: Cardiovascular disease
BP: Blood pressure
SBP: Systolic blood pressure
DBP: Diastolic blood pressure
BMI: Body mass index
FPG: Fasting blood glucose
TC: Serum total cholesterol
TG: Triglyceride
HDL-C: Serum high density lipoprotein cholesterol
COR: Crude odds ratio
AOR: Adjusted odds ratios
CI: Confidence intervals
DIU: Diuretics
ACEI: Angiotensin-converting enzyme inhibitors
ARB: Angiotensin receptor blockers
BB: β-blockers
CCB: Calcium channel blockers.
