# Supplementary material for: Analysis on associated factors of uncontrolled hypertension among elderly hypertensive patients in Southern China: a community-based, cross-sectional survey
Source: BMC Public Health. 2014 Sep 2;14:903. doi: 10.1186/1471-2458-14-903 (PMC4247067; doi:10.1186/1471-2458-14-903)
Supplement: Supplementary file 1 — Additional file 1: Distribution of socio demographic factors among participants, by clusters (n = 10644). (DOC 42 KB) [file 12889_2014_7294_MOESM1_ESM.doc]

**Additional file 1- Distribution of socio demographic factors among participants, by clusters (n=10644)**

| **Variables** | **Urban (n=3010)** | **Suburban (n=4481)** | **Rural (n=3153)** | **P-value** |
| --- | --- | --- | --- | --- |
| **Men, n (%)** | 1468 (48.8) | 2144 (47.8) | 1505 (47.7) | 0.42 |
| **Age (years)** | 72.2±8.1 | 71.0±8.2 | 71.4±8.0 | 0.06 |
| **BMI (kg/m2)** | 23.7±3.0 | 24.2±3.3 | 23.4±2.8 | **0.21** |
| **Education, n (%)** |  |  |  |  |
| College or higher | 304 (10.1) | 53 (1.2) | 30 (1.0) | **<0.001** |
| Middle | 1285 (42.7) | 232 (5.2) | 190 (6.0) |
| Primary | 1029 (34.2) | 1793 (40.0) | 1391 (44.1) |
| Illiterate | 392 (13.0) | 2403 (53.6) | 1542 (48.9) |
| **Family history of hypertension, n (%)** | 536 (17.8) | 811 (18.1) | 599 (19.0) | 0.44 |
| **Smoker, n (%)** | 513 (17.0) | 851 (19.0) | 582 (18.5) | 0.09 |
| **Alcohol intake, n (%)** | 501 (16.6) | 759 (16.9) | 490 (15.5) | 0.25 |
| **Overweight, n (%)** | 903 (30.0) | 1340 (29.9) | 905 (28.7) | 0.44 |
| **Obesity, n (%)** | 300 (10.0) | 339 (7.6) | 207 (6.6) | **<0.001** |
|  | | | | |
